# Methodological challenges in translational drug response modeling in cancer: A systematic analysis with FORESEE

**DOI:** 10.1371/journal.pcbi.1007803

**Published:** 2020-04-20

**Authors:** Lisa-Katrin Schätzle, Ali Hadizadeh Esfahani, Andreas Schuppert

**Affiliations:** 1 Joint Research Center for Computational Biomedicine, RWTH Aachen University, Aachen, Germany; 2 Aachen Institute for Advanced Study in Computational Engineering Science, RWTH Aachen University, Aachen, Germany; UNITED STATES

## Abstract

Translational models directly relating drug response specific processes that can be observed *in vitro* to their *in vivo* role in cancer patients constitute a crucial part of the development of personalized medication. Unfortunately, current studies often focus on the optimization of isolated model characteristics instead of examining the overall modeling workflow and the interplay of the individual model components. Moreover, they are often limited to specific data sets only. Therefore, they are often confined by the irreproducibility of the results and the non-transferability of the approaches into other contexts. In this study, we present a thorough investigation of translational models and their ability to predict the drug responses of cancer patients originating from diverse data sets using the R-package FORESEE. By systematically scanning the modeling space for optimal combinations of different model settings, we can determine models of extremely high predictivity and work out a few modeling guidelines that promote simplicity. Yet, we identify noise within the data, sample size effects, and drug unspecificity as factors that deteriorate the models’ robustness. Moreover, we show that cell line models of high accuracy do not necessarily excel in predicting drug response processes in patients. We therefore hope to motivate future research to consider *in vivo* aspects more carefully to ultimately generate deeper insights into applicable precision medicine.

## Author summary

In the context of personalized medicine, finding genomic patterns in a cancer patient that can predict how a specific drug will affect the patient’s survival is of great interest. Translational approaches that directly relate drug response specific processes observed in cell line experiments to their role in cancer patients have the potential to increase the clinical relevance of models. Unfortunately, existing approaches are often irreproducible in other applications. In order to address this irreproducibility aspect, our work comprises a thorough investigation of a diverse set of translational models. In contrast to other approaches that focus on one isolated model characteristic at a time, we examine the overall workflow and the interplay of all model components. Additionally, we validate our models in multiple patient data sets and identify differences between cell line and patient models. While we can establish models of high predictive performance, we also expose the deceptive potential of optimizing methods to a specific use case only by showing that those models do not necessarily depict biological processes. Thus, this study serves as a guide to interpret new approaches in a broader context to avoid the dissemination of noise-driven models that fail to serve in everyday applications.

## Introduction

Within the context of a permanently growing interest in precision medicine over the last years, where therapies are intended to be tailored to specific characteristics of individual patients, the study of drug sensitivity prediction for complex diseases, such as cancer, has experienced a tremendous boost [1]. The availability of both the computational power to work with complex algorithms and large-scale pharmacological data sets gave rise to various drug sensitivity studies. Since *in vitro* experiments are easily standardizable, readily quantifiable and feasible for high-throughput settings, cell lines moved up to become convenient test specimens to explore the characteristics of diverse diseases and the mechanisms behind drug action.

Thus, cell lines were not only extensively characterized by means of their molecular profile, such as mutational status, gene expression, proteomics, copy number variation or methylation, but also based on their responses to broad panels of drugs [2–7]. Consequently, a variety of computational approaches were developed to connect the disease-specific molecular profiles and drug responses [8–12]. In order to keep an overview of the versatile field of drug sensitivity prediction, efforts were also made to systematically compare existing approaches, for example in collaborative projects, such as the DREAM challenge by Costello et al. [13].

While these efforts are great in comparing and improving distinct components of a model, such as batch effect correction methods [14], feature selection methods [15] or regression algorithms [1], they often lack the consideration of the complete modeling workflow and the interplay of the individual pipeline components. Moreover, published methods are inclined to be biased towards the authors’ fields of expertise, which hinders a fair and objective benchmarking of existing methods. Consequently, a lot of different models exist in the literature, which are all carefully adapted for very specific purposes and distinct data samples, while generic rules for promising models are difficult to find.

Furthermore, in the majority of these studies, the ultimate goal is to understand the molecular mechanisms in living patients, while the analyses are performed on *in vitro* data. Even though studies have shown that cell lines reflect many important aspects of human *in vivo* biology, they also exhibit significant differences. This is especially evident regarding the absence of an immune system and a micro-environment of tumor cells in cell cultures, making the direct translation of preclinical models into a clinically relevant context rather difficult [16]. Translational models that relate *in vitro* and *in vivo* mechanisms already in the model building process [17], can help to tackle the discrepancy between abstract training of cell line-based models and their clinical application. One prominent solution is to address the genomic differences between *in vitro* and *in vivo* data via batch-effect removal concepts [18]. However, corresponding to the challenges that are opposing pure cell line models, translational models are often not robust across different patient data sets. Moreover, the processes that are relevant to the fitting often remain invisible.

Accordingly, in this study, we intend to systematically investigate the workflow of translational models of such kind, using our R-package FORESEE [19] to explore all relevant model components and their interplay. By including publicly available approaches and running the different modeling pipelines automatically, we reduce the bias introduced by the users’ heterogeneous experiences in those methods. Furthermore, we directly evaluate the resulting models on patient tumor data to inherently attain clinical relevance of our models. By addressing noise, sample size effects and a lack of drug-specificity in our models, we point out possible factors that can affect the robustness of a model. Thereby, we want to demonstrate how a model that seems to perfectly predict drug response in one scenario does not necessarily describe meaningful patterns that can help to advance the general understanding of drug response mechanisms. Instead, we want to encourage future investigations to dig deeper into understanding the disease and disease treatment models by broadening the validation of models, considering more and larger data sources and comparing more modeling pipelines. Moreover, we aim to show that pipelines that model cellular drug response mechanisms with high accuracy cannot automatically predict *in vivo* processes and therefore hope to motivate research to consider *in vivo* aspects more carefully to ultimately generate deeper insights into applicable precision medicine.

## Results

### A systematic scan of the model space reveals models of outstanding performance

**Fig 1:**
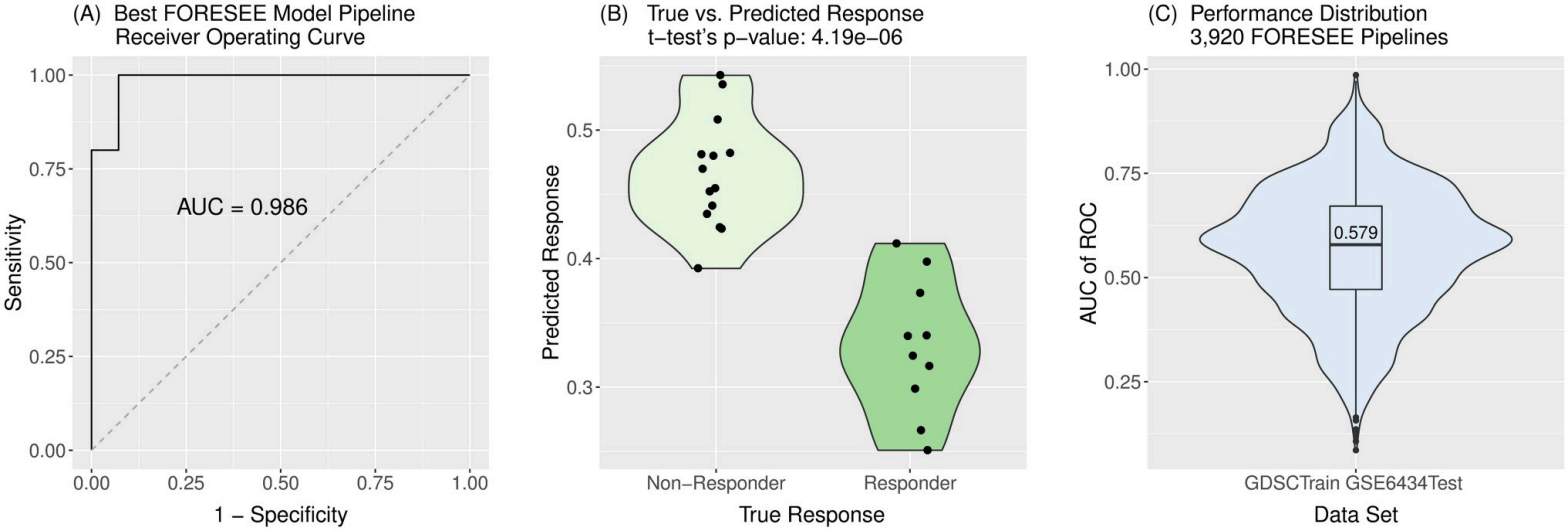
Performance portrayal of translational models trained on GDSC cell line data using the R-package FORESEE to predict GSE6434 patient drug response. Of a total of 3,920 modeling pipelines, the best modeling pipeline had the following settings: drug: Docetaxel, cell response type: ln(IC_50_), cell response transformation: binarization with k-means, sample selection: all, duplication handling: remove all duplicates, homogenization: remove unwanted variation, feature selection: landmark genes, feature preprocessing: none, black box algorithm: elastic net (while lasso would have yielded the same performance). (A) The receiver operating curve of the best model reveals an AUC of 0.986. (B) The comparison of the true responders and non-responders and their separation obtained from the best FORESEE model shows an almost perfect distinction, with a p-value of a t-test of 4.19e-6. (C) The performance distribution of all 3,920 model pipelines reveals a median AUC of ROC of 0.579.

In order to systematically scan the space of translational models, the FORESEE package was applied to different combinations of cell line- and patient data sets, combining five methods of cell response preprocessing with seven approaches of homogenization and batch effect correction, four kinds of feature selection, four ways of feature preprocessing and seven model algorithms. The scan of these 3,920 different modeling pipelines revealed models of very high performance. Especially for the breast cancer patient data set GSE6434 [20], multiple good model pipelines could be found, with 189 of them yielding an AUC of ROC > 0.81, which is the reported performance of the established translational ridge regression model by Geeleher et al. [18]. The best model pipeline, implementing a k-means binarization of the reported IC_50_ values, a remove unwanted variation (RUV) homogenization between the cell line- and patient data set, a landmark gene filter, no further feature preprocessing of the remaining gene expression features, and an elastic net or a lasso regression algorithm, which both accomplished a performance of AUC of ROC = 0.99 (Panel A in Fig 1), produced a near to perfect separation between responders and non-responders of the treatment (Panel B in Fig 1). Still, the 3,920 different modeling pipelines manifested a high heterogeneity with a wide performance distribution (Panel C in Fig 1) and a median AUC of ROC of 0.579. As portrayed in S1 Fig, each of the other patient data sets shows a similarly wide-spread performance distribution for the 3,920 models tested with FORESEE. Yet, models of very high performance can be found for each of them, the best model settings being listed in Table 1.

**Table 1:** Model performance and pipeline settings of the best model for each patient data set.

Data Set	Cell Response Transformation	Homogenization	Feature Selection	Feature Preprocessing	Black Box Algorithm	AUC of ROC	AUC of PR	p-value of t-test
**GSE6434**^(a)^ **Docetaxel**	binarization k-means	RUV	landmark genes	none	elasticnet	0.986	0.655	4.19e-06
**GSE6434**^(b)^ **Docetaxel**	binarization k-means	RUV	landmark genes	none	lasso	0.986	0.655	1.08e-05
**GSE18864 Cisplatin**	logarithm	none	landmark genes	zscore genewise	rf	0.822	0.307	0.0216
**GSE51373 Paclitaxel**	powertransform	RUV4	all	none	rf	0.859	0.307	1.03e-03
**GSE33072 Erlotinib**	powertransform	RUV4	all	none	rf	0.897	0.307	7.54e-04
**GSE33072 Sorafenib**^(a)^	binarization k-means	YuGene	p-value	none	linear regression	0.697	1	0.0406
**GSE33072 Sorafenib**^(b)^	binarization cutoff	RUV4	landmark genes	none	rf	0.697	1	0.0406
**GSE9782 GPL96 Bortezomib**	none	ComBat	landmark genes	PCA	svm	0.680	1	6.76e-05
**GSE9782 GPL97 Bortezomib**	binarization k-means	limma	p-value	PCA	rf	0.704	0.714	4.95e-06

^(a,b)^ Two models having the same (best) performance value for the respective data set.

All models were trained on ln(IC_50_) values of the respective drug.

All models were trained on all available GDSC cell line samples in the training data set without tissue specifications.

All models removed all feature names occurring more than once from the training process.

### Model performance is patient data specific

#### A perfect model for a specific data set is insignificant for other use cases

**Fig 2:**
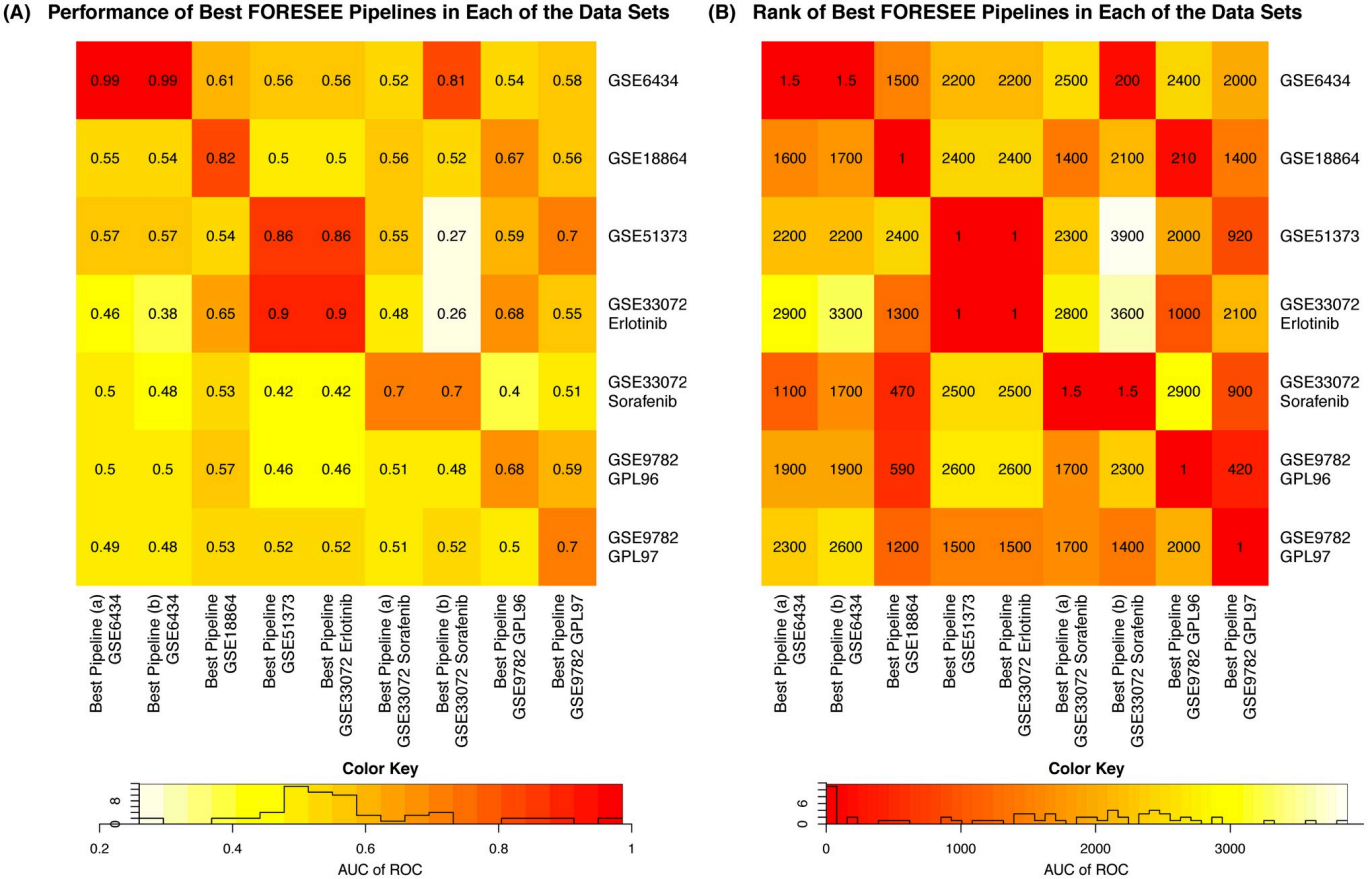
Heatmaps of the performance of the best modeling pipeline of each patient data set in each of the other patient data sets. (A) The color depicts the AUC of ROC of the respective pipelines. (B) The color represents the rank of the modeling pipeline among all 3,920 pipelines that were trained for a specific data set. More details of the modeling pipelines are listed in Table 1. The ranks of the best pipelines of the GSE6434 data set and the Sorafenib subset of the GSE33072 data set are 1.5, as two modeling pipelines yielded the exact same performance for the respective data set.

From the comparison of the settings of the best model in each of the cell line—patient data set scenarios in Table 1 it becomes apparent that the best model pipeline settings vary strongly between the different patient data sets. One specific modeling pipeline that yields the best performance in all of the data sets does not exist. For a visual representation of this finding, Fig 2A shows the AUC of ROC of the best model of each of the analyzed patient data sets (Table 1) and the performances of the same models, when they are used to predict the other patient data sets, in a colored heatmap. Since there were two modeling pipelines that yielded the best performance in the GSE6434 data set (AUC of ROC = 0.99) and two for the Sorafenib subset of the GSE33072 data set (AUC of ROC = 0.7), nine pipelines are compared with respect to their performance in the seven patient data sets. Fig 2B depicts the same information as Panel A, but in ranks rather than absolute numbers. It becomes evident that a model that yields almost perfect prediction performance in one of the data sets has a merely mediocre performance for other data sets. For example, one of the best modeling pipelines (GSE6434 (a)) that was found for modeling the Docetaxel response of GSE6434 patients—the elastic net regression model that was trained on k-means binarized IC_50_ values of GDSC cell lines and RUV homogenized gene expression values of the set of landmark genes without any further preprocessing of the features—yields an AUC of ROC = 0.99 in this setting, while for the Cisplatin response of GSE18864 patients the same model only generates an AUC of ROC = 0.55, which is ranked 1,600th among all 3,920 pipelines. One modeling pipeline however constitutes an exception: the model trained on power-transformed ln(IC_50_) values, with RUV4 as homogenization method between training and test features, including all genes as features without further feature preprocessing and a random forest algorithm for model training yields the best performance of all 3,920 modeling pipelines in both the GSE51373 data set (AUC of ROC = 0.859) and the Erlotinib subset of GSE33072 (AUC of ROC = 0.897). Despite this outstanding performance in two of the data sets, the pipeline fails to exceed an AUC of ROC of 0.56 in all other data sets, confirming the more general notion that a perfect model for a specific data set is insignificant for other use cases.

#### Model performance is poorly correlated among patient data sets

From our systematic scan of model space, it becomes clear that the problem of reproducibility does not only occur for the very best pipeline in each specific data set, as shown in the previous section. This problem manifests for almost all modeling pipelines, independent of the absolute value of their performances.

**Fig 3:**
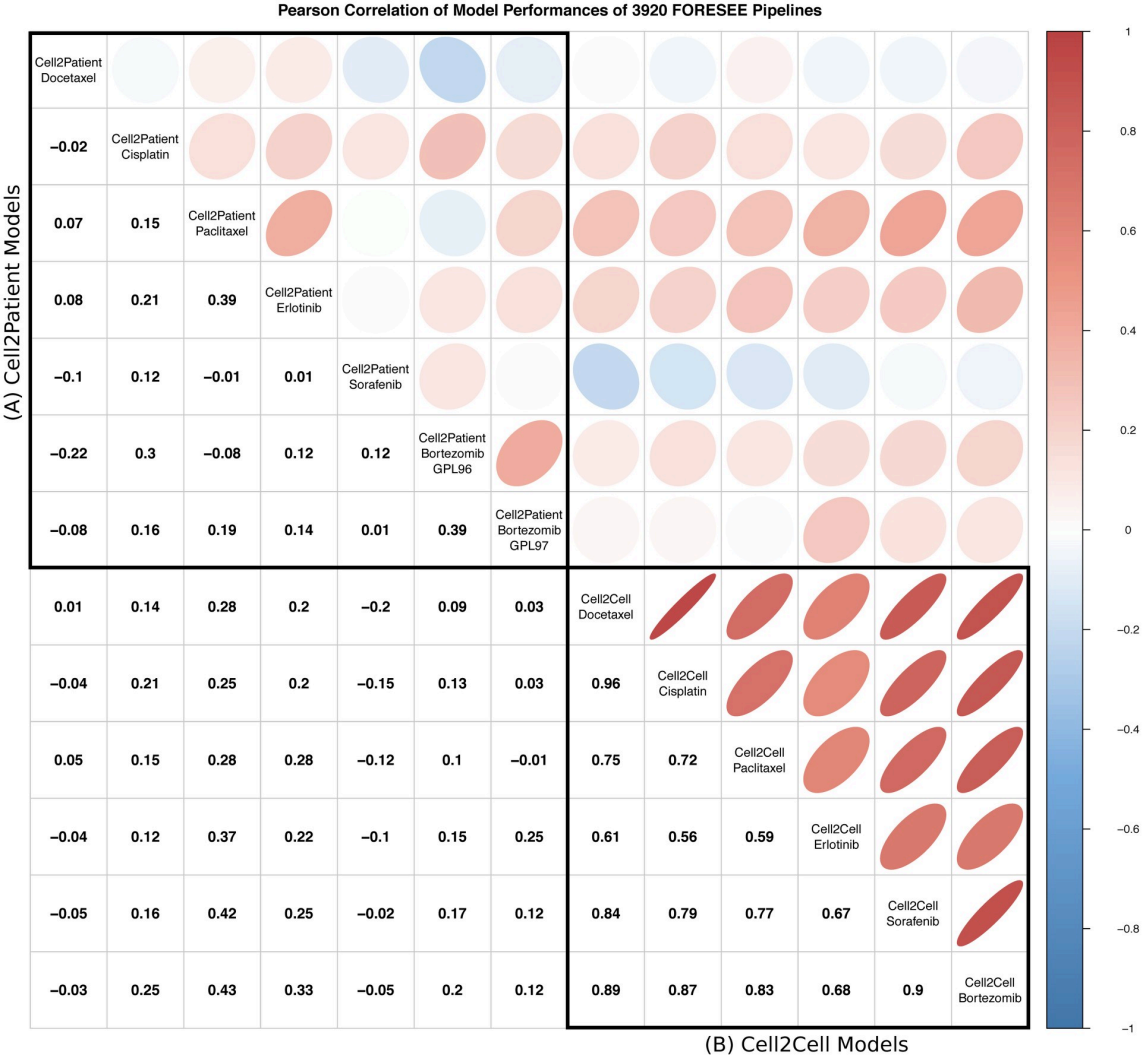
Pearson correlation of the performances of 3,920 FORESEE pipelines among the different cell line- and patient data sets. Pearson correlation of the performances of 3,920 FORESEE pipelines for seven different patient data sets (A): GSE6434, GSE18864, GSE51373, GSE33072 Erlotinib cohort, GSE33072 Sorafenib cohort, GSE9782 GLP96 cohort and GSE9782 GLP97 cohort and (B) six different cell line model scenarios with data from GDSC for Docetaxel, Cisplatin, Paclitaxel, Erlotinib, Sorafenib and Bortezomib. The performance measure for the cell line-to-cell line modeling scenarios is the mean AUC of ROC of a 5-fold cross-validation for each drug.

Section A in the upper left corner of the correlation plot in Fig 3 portrays the Pearson correlation of the AUC of ROC performance values of 3,920 modeling pipelines in different patient data sets. Not only are the absolute correlation values very low, but also some of the patient data set pairs exhibit a negative correlation. Especially the correlation between the GPL96 and the GPL97 cohort of the GSE9782 data sets, which comprises the gene expression data of the exact same patients only measured with two different array technologies, exposes a surprisingly low value of 0.39. Notably, in the context of generally rather low correlation values, the correlation of 0.39 between GSE51373 and the Erlotinib cohort of GSE33072, which also share the same best performing modeling pipeline (Fig 2A and 2B), appears to be exceptionally high. Still, the overall mediocre correlation implies that it is impossible to reliably determine if a certain modeling pipeline will yield a good (or bad) prediction performance in another data set, even though the performance in one of the data sets is known.

In order to put the poor reproducibility into perspective and examine the problem in a broader context, S2 Fig features the conditions of the underlying data sets that were used in the presented analyses. After all, there are immediate factors that affect the model performance that do not originate from model specifications.

First, even though all of the models were trained on cell line data from GDSC, the training data sets differed enormously. This is owed to the fact that the drugs included in the GDSC database are not screened on all of the available cell lines, but mostly on smaller subsets. Consequently, the training data sets for the different translational scenarios in this paper varied in sample size and in the distribution of the cell lines’ tissues of origin depending on the drug that was modeled (S2C Fig).

Second, the features that were available for training the translational model varied significantly. Both, the microarrays that were used in the generation of the patient gene expression data and the gene IDs that were originally chosen to define the features, have an impact on the resulting overlapping feature list for each distinctive pair of cell line- and patient data set. After converting the reported gene names into Entrez Gene IDs [21] with biomaRt [22, 23] and removing all duplicates, the feature set size varied from 4,786 genes in translational models for the GSE9782 GPL97 data set to 15,703 genes in translational models for the GSE33072 Erlotinib and Sorafenib data sets before any feature selection method was applied. As a consequence, all of the gene filter methods *none*, *variance* and *pvalue* produced feature sets that could differ in size by a factor of 3, which makes the comparison of these methods in two different data sets unbalanced.

Third, while the training sets’ distributions of gene expression values were highly similar in all seven settings (S2A Fig)—which is expected, since all training sets are subsets of the same cell line database GDSC [2]—the distribution of gene expression differed enormously between the different patient data sets (S2B Fig). For this very reason, the homogenization of the training and test data set pairs is a highly important element of a modeling pipeline. The common homogenization methods should be well prepared for these differences, whereas the option *none*, which simply skips any type of homogenization between the training and test set, can result in very different conditions for different patient data sets. Consequently, it is no surprise that some of the pipelines do not show a high correlation among the different patient data sets.

Taking into account these effects of the data constitution, it becomes accessible that the correlation of the ensemble of 3,920 pipelines is not in a perfect range. Still, they do not justify the large extent of the bad correlation that was attained in this study and the lack of transferability of these approaches in general.

### Model performance is affected by noise

Concluding from the weak correlation of model performances among different data sets and the widespread performance distributions of the set of 3,920 pipelines in one individual data set, the translational models that are investigated in this study seem to be affected by noise. In order to assess the extent of this noise, we investigated three different aspects that could potentially compromise modeling performance.

#### The sample size of the patient data sets affects the perception of noisy results

As apparent from the violin plots in Fig 1 and S1 Fig, models seem to yield much higher performances for data sets such as GSE6434 with a maximum AUC of ROC of 0.986 than data sets such as the GSE9782 GPL97 cohort with a maximum AUC of ROC of only 0.704. While there is no doubt that noise varies from data set to data set, such that certain ones are better adapted for model training than others, it is still reasonable to investigate the circumstances of this performance disparity in more detail.

**Fig 4:**
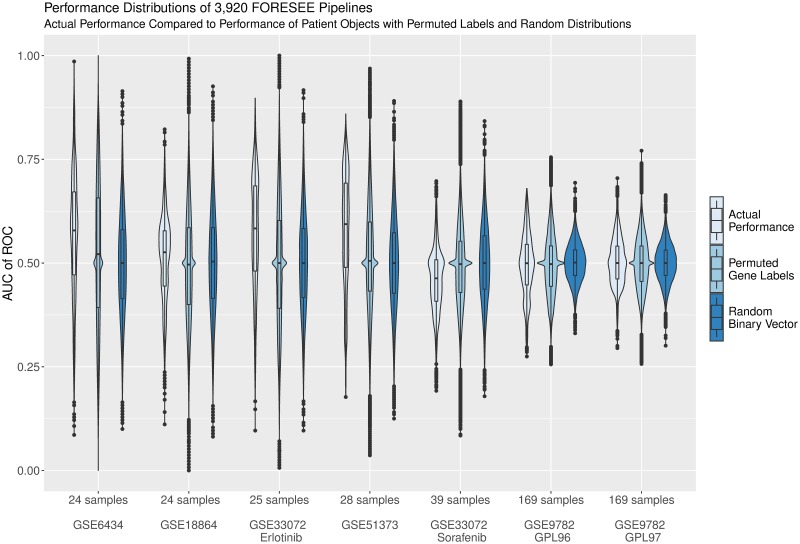
Violin plots of the FORESEE performances compared to random distributions. Distributions of the performances of all 3,920 FORESEE modeling pipelines in each of the seven different patient data sets: GSE6434, GSE18864, GSE51373, GSE33072 Erlotinib cohort, GSE33072 Sorafenib cohort, GSE9782 GLP96 cohort and GSE9782 GLP97 cohort. The actual performance distributions of the translational models (light blue) are compared to the distributions, where each of the 3,920 translational models was applied to 1,000 patient objects with randomly permuted gene labels (medium blue) and to distributions, where the actual patient response values are compared to 10,000 randomly generated binary vectors to calculate an artificial AUC of ROC measure (dark blue). Data sets are shown in increasing sample size from left to right.

One principal difference between the patient data sets is their sample size. Therefore, Fig 4 displays the performance distributions of all 3,920 modeling pipelines in the order of increasing sample size of the patient data sets (light blue violin plots). Following the principle that it is a lot more probable to correctly guess the responses of a small sample set than those of a very large sample set at random, it becomes explicit that data sets that exhibit a wide spread of performance values with high reaching peak performances have a significantly smaller sample size (GSE6434: 24 patients, GSE18864: 24 patients and GSE51373: 25 patients) than data sets with a narrow performance distribution (GSE9782 GPL96 and GPL97: 169 patients). For the purpose of illustration of this sample size effect, Fig 4 includes distributions that represent artificial AUC of ROC values from the comparison of 10,000 randomly generated binary vectors in the size of the patient data sets compared to the actual responses of the respective patient data sets (dark blue). Moreover, Fig 4 depicts distributions that summarize the performances of the 3,920 FORESEE model pipelines applied to 1,000 versions of each patient object, where the gene labels were randomly permuted (medium blue).

From the direct comparison between the actual performance distributions and the random ones, it becomes apparent that certain model findings may be less significant than previously thought. In the cases of GSE9782 GPL96 and GPL97 for instance, even though the best models have comparably low absolute values of AUC of ROC, their performances are still higher than predictable by 10,000 random guesses. Thus, they effectively allow the training of robust models that are not entirely driven by noise. The Sorafenib cohort of the GSE33072 data set and the GSE18864 data on the other hand yield worse results on average when a model is trained to predict the response than when the response is guessed at random, even though the absolute values of the AUC of ROC seem more promising than the ones of GSE9782 GPL96 and GPL97. This is particularly prominent for the Sorafenib cohort of the GSE33072 data set, which exhibits a median AUC of ROC of 0.46—the lowest among all data sets.

This indicates that these data sets are predominantly governed by noise and should therefore be considered with caution for the training of universally valid clinical models. For GSE18864, this finding is concurrent with previous findings by Geeleher et al. [18], whose models could not capture variability in clinical response, and by Silver et al. [24], the authors of the original study, who could not identify a predictive gene signature of Cisplatin response. Similarly, a unified set of biomarker genes that establishes a robust prediction of Sorafenib responses for the GSE33072 data set has not been determined so far [25].

Even though, compared to GSE18864 and the Sorafenib subset of GSE33072, the 3,920 FORESEE models yield a better performance distribution in the other data sets, it becomes explicit that the fitted models do not outperform the randomized models by far. Especially the large range of the performance distribution of translational models applied to patient data with permuted gene labels (medium blue) demonstrates that an optimized model of very high predictive performance in one application scenario—as listed in this study or as published separately by other groups—does not necessarily produce a model that truly captures the mechanisms that are relevant for the drug response and can therefore not be employed in clinical practice. Instead, the high predictivity can equiprobably stem from random mechanisms that were captured by the model by chance and are only relevant to that specific scenario. In this sense, our analysis demonstrates the importance of considering the possible degree of randomness that could affect modeling results, especially when the sample size of the test population is small.

#### Translational models are not necessarily specific to the drug of interest

In an attempt to further analyze the extent of randomness accompanying the performance results of the shown FORESEE modeling pipelines, a drug specificity analysis was conducted. For each of the patient data sets and each of the 266 drugs contained in the GDSC database, a set of 100 randomly chosen pipelines, which are listed in S1 Table, was used to train translational models on the GDSC cell line data and predict the respective patient response.

**Fig 5:**
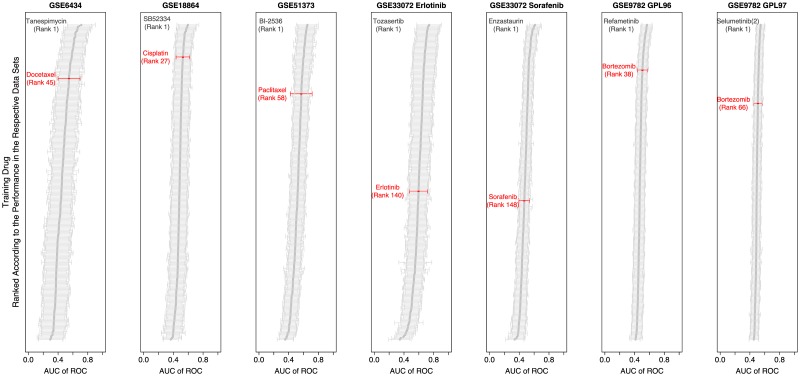
Drug specificity plot of FORESEE modeling pipelines. Impact of the training drug on model performance for each of the seven different patient data sets: GSE6434, GSE18864, GSE51373, GSE33072 Erlotinib cohort, GSE33072 Sorafenib cohort, GSE9782 GLP96 cohort and GSE9782 GLP97 cohort. A set of 100 pipelines, which are listed in S1 Table, were randomly chosen and used to train translational models on the GDSC cell line data with each of the 266 drugs contained in the GDSC database individually and then tested on each of the patient data sets. For each of the data sets, the drugs are ordered with respect to the mean AUC of ROC of the 100 random pipelines trained with that drug. The red color marks the drug that is actually applied to the patient. The first-ranked drug is additionally indicated in order to facilitate the comparison of the different drugs and their modes of action. As an exception, for predicting GSE9782 GPL97 patient outcome, six pipelines that include RUV as homogenization method were not trained on those drugs that resulted in a training set that had more samples than features, as this was not compatible with the PCA step performed in this method.

Fig 5 depicts the drugs that were used during the training process for each individual patient data set ranked according to their mean performance of 100 modeling pipelines. In none of the cases the drug that is administered to the patients is the best option to pick for training the translational models on the cell line data. While for the majority of the data sets—the Docetaxel treated breast cancer patients of GSE6434, the Cisplatin treated breast cancer patients of GSE18864, the Paclitaxel treated ovarian cancer patients of GSE51373, and the Bortezomib treated patients with multiple myeloma of GSE9782—the specific drug that is administered to the patients is in the top range of training drugs, for other data sets, namely the patients of GSE33072 with non-small cell lung cancer that were treated with Erlotinib or Sorafenib, the drugs of interest only yield performances in the lower half of the list of drugs investigated.

To a certain degree, it is expectable that drugs that differ in their specific mode of action still kill the same cancer cells and therefore have a comparable effect on cancer patients, because drug action is also impacted by general characteristics, such as unspecific cell death and survival mechanisms, drug efflux or resistance properties. Still, especially in the context of searching for distinct drug targets and related fields, such as drug repurposing, the specific mechanisms of drug action are considered a major contribution towards the cell’s or the patient’s survival in the end. Yet, especially the examples of Erlotinib and Sorafenib in this analysis demonstrate that the effect of the drug that is used during the training process on model performance is merely minor. Indeed, the fact that so many models reveal good performances, even though the drugs that the models were trained with are not the ones that were predicted in the end, suggests that the internal mechanisms that dominate the translational models for drug action in cancer patients are not at all specific to the drug of interest, but rather to general mechanisms of drug response. This hypothesis is further supported by the observation that the drugs that are more predictive than the drugs of interest do not share the same mode of action. For example, Docetaxel is a chemotherapeutic drug that binds to microtubules and prevents their disassembly, eventually causing the initiation of apoptosis, while the drug occupying the first rank in predicting GSE6434 patients, Tanespimycin, is an antineoplastic antibiotic that inhibits the heat shock protein 90 (Hsp90), which then promotes the proteasomal degradation of oncogenic signaling. Likewise, Erlotinib is an inhibitor of EGFR signaling, while the drug that is ranked first for GSE33072 patients is Tozasertib, which targets aurora kinases.

#### The lack of model transferability is not endorsed by *in vitro* models

Following the observation that randomized models can yield prediction performances close to performances gained from actually trained translational models and the finding that translational models are not necessarily specific to the administered drug, the question arises whether the 3,920 modeling pipelines tested in this study are at all capable of predicting drug response or if all observations should be attributed to noise. In a baseline experiment, we therefore investigated *in vitro* models that were trained and tested on cell line data in a five-fold cross-validation for all six drugs of interest: Docetaxel, Cisplatin, Paclitaxel, Erlotinib, Sorafenib and Bortezomib. In order to keep the cell line models comparable to the translational patient models, the features used for training the *in vitro* models were restricted to those that were available for the respective patient models. The violin plots in S3 Fig summarize the performances of 3,920 modeling pipelines for each drug. Moreover, section B in the lower right corner of the correlation plot in Fig 3 depicts the correlation of the set of cell line models for different drugs. With a minimum of 0.56 between Cisplatin and Erlotinib, and a maximum of 0.96 between Cisplatin and Docetaxel, the Pearson correlation for models that are trained and tested on cell line data is high enough to manifest that more than noise is fitted by the 3,920 modeling pipelines of the FORESEE routine and that the models capture a reasonable amount of information about drug response. While S3 Fig reveals a strong variation in the absolute values of model performance among different drugs, the correlation in Fig 3 proves that there are in fact groups of pipelines that yield consistently high and other groups that yield consistently low prediction performance for multiple drugs. Interestingly, the performance variation among different drugs for cell line models is not the same as the variation observable for different translational patient models. While the distribution of model performances for cell line models for Erlotinib resides around an AUC of ROC of 0.57, for example, which is the lowest of all tested drugs, the set of translational patient models for Erlotinib shows a median of 0.58, which makes it one of the best three translational model scenarios. Taken together, this attenuates the potential speculation that the differences observed for modeling different drugs reside purely on the extent to which drug mechanisms of a specific drug candidate can be captured within a gene expression profile. Instead, the results of the *in vitro* study point towards the notion that information about drug response and the cell death or cell survival mechanisms can in fact be captured in the underlying gene expression data and can be used to create a prediction model for all investigated drugs in this study. Consequently, the lack of transferability between the different translational models reveals that there are other, more complex challenges to overcome for the translation from *in vitro* processes into an *in vivo* setting.

### Simple model settings improve translational model performances

As shown in the previous analyses, there is no modeling pipeline that yields outstanding prediction performance for all data sets that were investigated. Yet, it is possible to identify settings that help to generate more promising models.

#### An enrichment analysis reveals that simpler translational models yield higher performances

**Fig 6:**
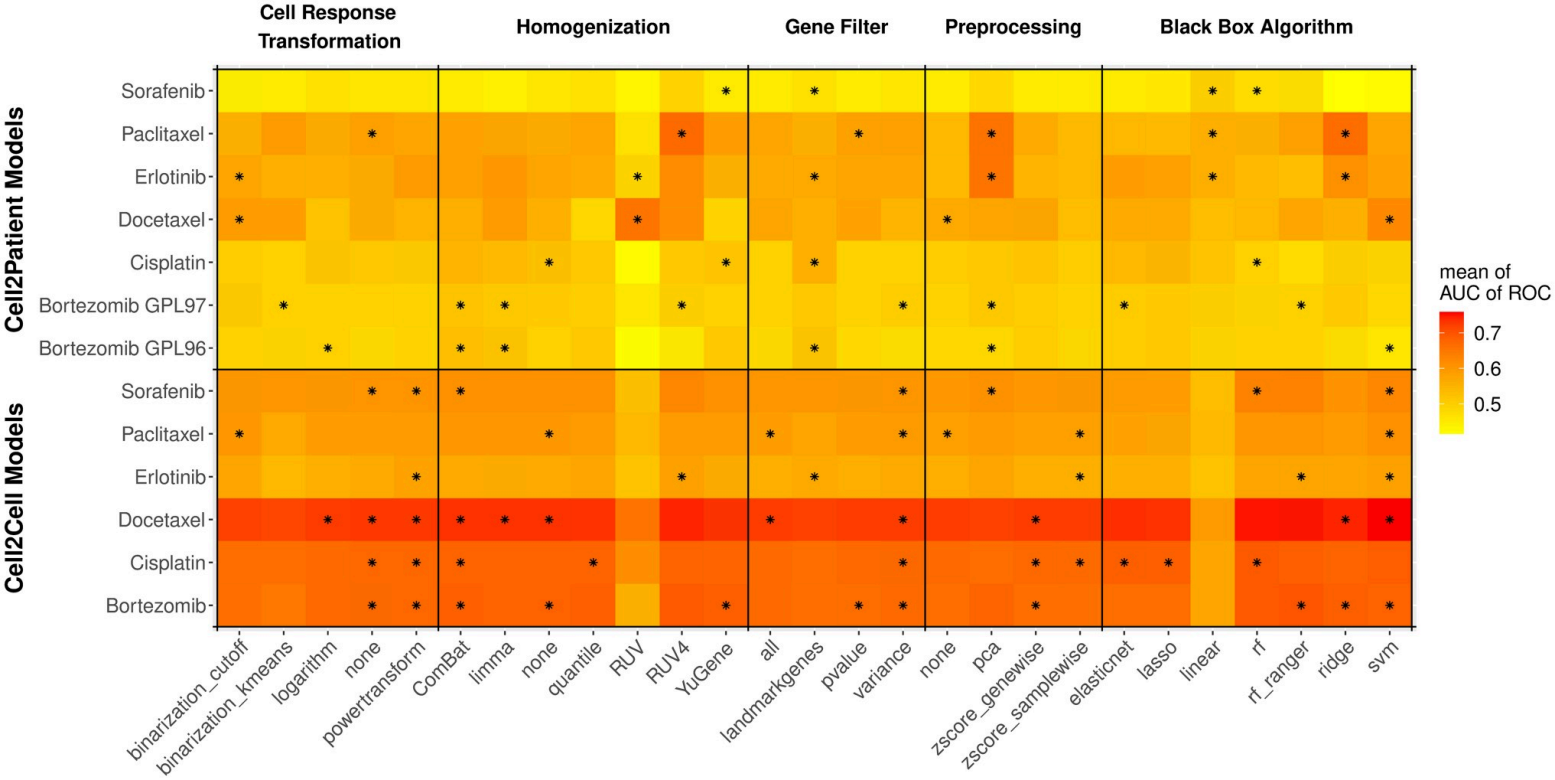
Heatmap of the performances of 3,920 FORESEE pipelines in the different cell line- and patient data sets averaged for model setting categories. Heatmap of the performance of 3,920 FORESEE modeling pipelines tested with seven different patient data sets (*Cell2Patient*): GSE6434, GSE18864, GSE51373, GSE33072 Erlotinib cohort, GSE33072 Sorafenib cohort, GSE9782 GLP96 cohort and GSE9782 GLP97 cohort, and six different cell line model scenarios (*Cell2Cell*): GDSC data for Docetaxel, Cisplatin, Paclitaxel, Erlotinib, Sorafenib and Bortezomib. The performance measure for the cell line-to-cell line modeling scenarios is the mean AUC of ROC of a 5-fold cross-validation for each drug. The color depicts the mean AUC of ROC of modeling pipelines that comply with the corresponding model pipeline setting (x-axis). The black stars denote if a model pipeline setting is significantly enriched (*p* < 0.01) in the best 5% of all 3,920 modeling pipelines.

A closer investigation of which pipeline settings were enriched among the best model performances for each individual data set provided important insights into promising modeling choices. The top panel of Fig 6 summarizes the significantly enriched model settings in the best 5% of all 3,920 FORESEE pipelines in each of the patient data sets partitioned into the five varying pipeline categories: cell response transformation, homogenization, gene filtering, feature preprocessing and black box algorithm. Similar to the comparison of the best FORESEE modeling pipeline in each patient data set in Table 1, the significantly enriched model settings vary among the different data sets. Yet, some regularities can be observed for the best performing modeling pipelines:

- Binarization of the cell response, using either a cutoff value or the k-means algorithm, is beneficial in three of the seven data sets.
- Reducing the features to genes of the landmark gene list of the LINCS consortium during feature selection is significantly enriched in four of the seven data sets.
- Reducing the dimensionality of the input data by applying principal component analysis to the gene expression values is advantageous in four of the seven data sets.
- There are several well-performing black box algorithms, but linear regression is significantly enriched in three of the seven data sets.
- While RUV homogenization yields outstandingly high-performance values for the GSE6434 data set, it is strongly underperforming in every other data set.

Taking into account all categories together, it becomes explicit that settings that aim to simplify the model, such as binarizing the output data or reducing the dimensionality of the input data, tend to be significantly enriched in well-performing models and are therefore good principles to aim for.

#### Superior model settings for *in vitro* models differ from superior model settings for translational models

The overview provided by Fig 6, which lists the mean performance of every possible FORESEE model setting in this study as well as the enrichment in the best subset of model options for all data sets, allows for a direct comparison between *in vitro* models and translational models. Strikingly, model settings that work significantly better for *in vitro* models, such as support vector machine regression as modeling algorithm, no transformation or power-transformation of the cell line response values (ln(IC_50_)), z-score transformation of the gene expression values or a gene filter based on variance, do not show superiority for translational models that aim to predict *in vivo* drug performance. This finding is confirmed by another independent study, in which we compared the model performances of *in vitro* models and translational models. In order to investigate whether models that are proven to perform well for a certain set of cell lines also perform well on another set of cell lines, as well as on patients, for each drug of interest we split the GDSC data into three parts—a training set, a validation set and a test set—and compared the performance of 3,920 FORESEE modeling pipelines on the validation set, the test set and the patient data. The boxplots in S4 Fig depict that the set of 300 pipelines that perform best on the validation bin of the *in vitro* data also performs significantly better on the test bin of the *in vitro* data, proving that the information that is captured in those *in vitro* models can be applied to other *in vitro* use cases. If these 300 FORESEE pipelines of high performance are used to predict the response of patients in a translational model setting however, they do not show a significant improvement in 5 of the 7 scenarios. Thus, models that are selected based on their performance on cell line data alone, do not necessarily excel in predicting the drug response of patients. Likewise, model settings that yield higher prediction performance in translational models, such as a gene filter based on the landmark gene list or principal component analysis as feature preprocessing method, do not seem to be advantageous for *in vitro* models. In the case of linear regression, which is significantly enriched in the modeling of 3 out of 7 patient data sets, the performance in cell line data is even lower than every other modeling algorithm tested *in vitro*. Consequently, also the correlation between *in vitro* and translational models is rather low, as depicted in Fig 3. Hypothesizing based on these observations, it seems that drug-specific processes on the *in vitro* level are observable in a more isolated context, which allows to capture more details in more complex models, while these specific characteristics depreciate during the translation into the greater context of an *in vivo* setting, where drug response and survival processes are reflected in much simpler patterns.

#### Validation of model setting recommendations

**Fig 7:**
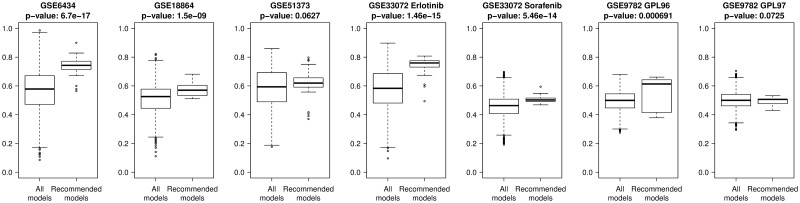
Validation of model setting guidelines. Boxplots of the performances of all 3,920 FORESEE modeling pipelines for each of the seven different patient data sets: GSE6434, GSE18864, GSE51373, GSE33072 Erlotinib cohort, GSE33072 Sorafenib cohort, GSE9782 GLP96 cohort and GSE9782 GLP97 cohort, compared to only those 35 pipelines that include landmark genes as gene filter, PCA as preprocessing method and linear regression as modeling algorithm. The p-values are the results of a t-test between the two distributions.

Despite the lack of transferability of both extremely high performing model pipelines and the general set of 3,920 pipelines with a poor correlation among different data sets, the enrichment analysis revealed that certain settings can be appointed that promote model performance. In order to validate those findings, we compared a subset of 35 modeling pipelines that followed the design rules established in this study—linear regression models fitted on genes that are part of the landmark gene list and preprocessed by applying PCA—to the overall set of 3,920 FORESEE pipelines. Fig 7 depicts the differences via boxplots, revealing that in 5 out of 7 data sets a significant improvement of model performance can be achieved if the design principles are applied. Only in GSE51373 and GSE9782 GPL97 the increase is not remarkable enough to be considered significant. Overall this confirms the hypothesis that simple models help to improve the performance of translational drug response prediction.

## Discussion

The ensemble of investigations of this study proves that a systematic analysis of translational models, not focussing on one specific model element only, but instead considering the whole modeling pipeline and the interplay of preprocessing methods and modeling algorithms, can be beneficial to refine the understanding of modeling coherences and key model characteristics. Tools, such as our R-package FORESEE, have the potential to support this systematic analysis by spanning an extensive space of model parameters that is searched for an optimal setting. By considering all combinations of model settings, our scan indeed spawned superior models for individual application scenarios. This superiority however was proven to not be robust across multiple data sets.

While this seems to resemble the common overfitting problem for models that are trained and tested on one data set only and fail to replicate in a new, unknown one, this finding is different in the sense that during the translational model fitting process, a model is trained on cell line data and it is not until after the training process that the model is applied to the patient data to obtain a prediction. Considered in isolation, this separation of training- and test set should provide for a definite quantification of model performance that is robust with respect to overfitting to the underlying data. Strikingly however, the incorporation of the search process on the full model parameter space seems to introduce an effect that is quite similar to the overfitting problem. In this regard, we could demonstrate that random predictions could in fact yield performance values that could compete with those of actual models. Moreover, we showed that the drug that a translational model is trained with does not have a prevailing impact on the model performance. Effectively, the training drug does not even have to share the same mode of action with the drug that is predicted to create a good model.

Taken as a whole, this proves that translational models in the fashion of the ones developed in this study are not yet able to explain all processes of drug action in patients. On the contrary, the processes that are dominating translational models are not yet understood, such that random or drug unspecific models seem to be similarly good predictors in most of the cases. Moreover, our study revealed that modeling pipelines that are well fitted to predict cell line drug response differ considerably from translational modeling pipelines that excel in predicting patient drug response. Yet, the concept of using *in vitro* experiments in the preclinical drug development stage to learn drug mechanisms and develop predictive models that are ultimately applied to patients is the current state-of-the-art in pharmacological research. With our analysis, we would therefore like to draw attention to the pitfalls that accompany this concept. While a computational model might capture perfectly the processes that are initiated during the drug response in cancer cell lines, this model might not be suitable to predict the therapeutic outcome of patients in the clinic. In order to find reliable *in vivo* predictors, regularities of other cases of drug applications in patients should be taken into account to establish an estimate of confidence for the approach.

One concept that transpires in the investigation of translational models in this study is the superiority of models that are kept simple by for example binarization of the training output data, feature reduction to landmark genes, input dimensionality reduction by PCA or application of simple regression methods. This phenomenon coincides with the KISS principle (keep it simple, stupid!), a well know principle in engineering and design, which promotes the avoidance of unnecessary redundancy and complexity in order to solve a problem. At the same time, our investigations demonstrate that the drug-specific processes in patients are not fully captured in simple translational models of the kind developed with FORESEE. Thus, besides keeping models simple and focussing on the acquisition of general processes of drug action and survival in the data, another promising approach could be to integrate profound and mechanistic knowledge about drug targets and patient-specific characteristics into the models.

Along the way to more insightful models, also other important factors should be considered; some of which also transpired within this study. One challenge in the development process of translational models is the extent of utilizable data. Despite the fact that the generation of omics data has experienced a tremendous boost over the last years, the availability of patient data sets that include both molecular data, such as gene expression, and a measure of drug efficacy is limited. Existing patient data sets comprise small sample sizes only, which makes model training with them susceptible to noise, as apparent from the sample size effect analysis. Moreover, these data sets are highly heterogeneous in terms of the used array technologies, the measured drug response type, the chosen gene names and the applied normalization method. The creation of robust models that are easily transferable to other data sets and into other contexts requires large data sets of the same structure and with the same preprocessing, such that they are effortlessly comparable. At the same time, models that are trained on data sets of small sample sizes only, should be thoroughly tested with regard to their randomness and robustness in more than one context before being purported as reliably predictive. To that effect, this study accentuates the importance of systematic and unbiased benchmarking of new approaches.

Another important aspect of the exploration of translational models is the reliability of the translational principle as such. With the great amount of noise and randomness in the results of current studies, more focus should be laid onto the investigation to what extent cell lines are actually predictive of patients and whether or not other model systems, such as organoids ([26]) or patient-derived xenografts ([27]), could be of higher similarity with respect to patients and therefore more suitable to understand drug mechanisms.

Lastly, missing predictivity of the translational models shown here could be partially ascribed to the molecular data type that the models were trained on. So far, the investigations of translational models were mainly focused on gene expression data of microarrays owed to the absence of patient data sets that comprise more than one *omics* data type and at least one measure of drug response. The inclusion of RNASeq data and other data types, such as mutational status, copy number variation, methylation and protein expression could significantly improve the predictive performance of models, simply by adding more levels of information. Since not all biomedical mechanisms are fully shown on the gene expression level, other data types could provide valuable insights to generate a detailed picture of the disease and drug mechanisms.

## Materials and methods

Analyses of this paper were conducted with the software *R* [31].

### Cell line data

For the training of the translational models in this study, both gene expression data and logarithmized IC_50_ (half maximal inhibitory concentration) values as drug response measure were derived from GDSC data [2] and formatted into a *ForeseeCell* object. The data base contains pretreatment gene expression data measured with Affymetrix Human Genome U219 arrays of 1,065 different human cancer cell lines stemming from diverse tissues.

### Patient data

For the testing of the translational models in this study, information of patients with breast cancer (GSE6434 [20] and GSE18864 [24]), lung cancer (GSE33072 [28]), ovarian cancer (GSE51373 [29]) and multiple myeloma (the Bortezomib arms of GSE9782 [30]) was organized into *ForeseePatient* objects including gene expression data and one measure of *in vivo* drug efficacy, which is summarized in Table 2. Details about the preparation of the data sets can also be found in the Supplementary File 2 of the FORESEE package [19].

**Table 2:** Patient data sets from GEO.

Data Set	Cancer Type	Drug	Samples	Responder/ Non-Responder	Treatment Status	Array
GSE6434 [20]	Breast Cancer	Docetaxel	24	10/14	Neoadjuvant Therapy	U95 Version 2*
GSE18864 [24]	Breast Cancer	Cisplatin	24	15/9	Neoadjuvant Therapy	U133 plus 2.0*
GSE51373 [29]	Ovarian Cancer	Paclitaxel	28	16/12	Treatment-Naïve	U133 plus 2.0*
GSE33072 [28]	Non-Small Cell Lung Cancer	Erlotinib	25	13/12	Treatment-Experienced	1.0 ST*
GSE33072 [28]	Non-Small Cell Lung Cancer	Sorafenib	39	20/19	Treatment-Experienced	1.0 ST*
GSE9782 [30]	Multiple Myeloma	Bortezomib	169	85/84	Relapsed**	U133A*
GSE9782 [30]	Multiple Myeloma	Bortezomib	169	85/84	Relapsed**	U133B*

*Affymetrix Human Genome

**1-3 Prior Therapies

### FORESEE

For the systematic comparison of different translational drug response modeling pipelines, the R-package FORESEE [19] was used. FORESEE, which is short for uniFied translatiOnal dRug rESponsE prEdcition platform, partitions the general modeling pipeline into defined functional elements in order to enable the user to thoroughly investigate the impact of each of them on the model performance. The FORESEE modeling routine comprises two major functions: the ForeseeTrain loop, which uses cell line data to train a translational model, and the ForeseeTest loop, which applies the learned model to new patient data and assesses its performance. During training, the cell response data is transformed to serve as model output, while molecular data is prepared to be used as model input: before the data is fed into a black box model, training samples are selected, duplicated features are removed, cell line- and patient data are homogenized by means of batch effect correction methods, and specific features are selected, transformed and combined. For model testing, the completed model is applied to molecular patient data that needs to be preprocessed in the exact same manner as the cell line data. The predictions can subsequently be compared to the reported patient drug responses to evaluate the model performance.

#### Cell response preprocessing

Five different methods to preprocess the cell response data were chosen. Three options kept the cell response values continuous in order to train regression models:

1 The method *none* used the reported cell response values without any preprocessing.
2 The method *logarithm* applied the natural logarithm to the response values. In case of negative drug response values, an offset equal to the negative minimum drug response value plus 1 was added to all response values in order to avoid negative arguments of the logarithm.
3 The method *powertransform* used the R-package car [32] to determine an exponent for a subsequent power transformation. Again, an offset equal to the negative minimum drug response value plus 1 was added to all response values, in case of negative drug response values.

Additionally, two different binarization methods were used for classification settings:

4 The method *binarization_cutoff* used the package bootnet [33] to split the drug response values at the median into two classes.
5 The method *binarization_kmeans* used the package Binarize [34] to define two classes of responders using k-means clustering.

#### Patient response preprocessing

While the patient data sets GSE6434 and GSE51373 already contained binary response annotations, all other patient data sets were binarized before usage to enable an effortless comparison. The GSE33072 data with Erlotinib and Sorafenib response were binarized by splitting the reported months of progression free survival at the median. For the patient data set GSE18864, patients, whose clinical response had been categorized as clinical complete response (cCR) or clinical partial response (cPR), were manually classified as responders, whereas patients, whose clinical response had been categorized as stable disease (SD) or progressive disease (PD), were classified as non-responders. Similarly, the responses to Bortezomib of the GSE9782 data set were manually classified into responders, if the response had been reported as complete response (CR), partial response (PR) or minimal response (MR), and non-responders, if no change (NC) or progressive disease (PD) had been reported.

#### Sample selection

Previous studies have shown an increased predictivity of translational drug response models whose training sets do not only include cell line samples from the same tissue of origin as the tumor, but also cell lines originating from diverse other tissues [18]. Therefore, all available cell line samples from various tissues were selected to train the models in this study.

#### Duplication handling

In order to provide unique features and avoid any mismatches, all gene names that occurred more than once were removed from both the training and the test object.

#### Homogenization

For the homogenization of the gene expression data and the batch effect correction between cell line train- and patient test data, seven different methods were chosen:

1. The method *ComBat* from the package sva [35] used empirical Bayes frameworks to adjust data for batch effects.
2. The method *limma* [36] removed covariate effects by fitting a linear model to the impact of the different batches.
3. In order to have a thorough investigation of the usefulness of homogenization methods, the option *none* offered to employ modeling pipelines without any correction.
4. The method *quantile* [37] homogenized the data of different origins with quantile normalization.
5. The method *RUV4* [38] homogenized the gene expression data sets with the help of a list of housekeeping genes [39], which are defined by a constant level of expression across tissues. In a singular value decomposition, these housekeeping genes were considered as negative controls to identify and subsequently remove unwanted variation.
6. The method *RUV* applied a self-implemented function that was inspired by the function *RUV4* [38] and a function by Geeleher et al. [18] in order to remove batch effects. The function *princomp()* of the *stats* package of R applied a principal component analysis on the gene expression of the housekeeping genes [39] of both data sets and determined the impact of unwanted variation by training a linear regression model with the function *lm()* of the *stats* package of R on the first 10 principal components. The residual gene expression of all genes was considered as homogenized data.
7. The method *YuGene* [40] applied a cumulative proportion approach to make the gene expression data sets comparable.

#### Feature selection

Selecting a smaller subset out of the thousands of genes in microarray data to increase the robustness of biological models is a widely studied topic. In this paper, we compare four simple approaches:

1. The method *landmarkgenes* reduced the features to the list of landmark genes that were determined as being informative to characterize the whole transcriptome [41].
2. The method *variance* used the function *var()* of the *stats* package of R to calculate the variance of each gene across all samples of the training data set. The 20% least variant genes were removed from the analysis.
3. The method *pvalue* calculated a student’s t-test with the function *t.test()* of the *stats* package of R between the most sensitive and the most resistant samples of the training data set. The 20% genes with the highest p-values were removed from the analysis.
4. The option *all* considered all overlapping genes between the train- and test object without further feature selection.

#### Feature preprocessing

For the standardization and transformation of the features, four different methods were chosen:

1. The method *zscore_samplewise* transformed the raw gene expression (GEX) value of each gene, by subtracting the mean GEX of all genes of one sample and subsequently dividing the resulting value by the standard deviation of the GEX of all genes of that sample.
2. The method *zscore_genewise* transformed the raw gene expression (GEX) value of each gene, by subtracting the mean GEX of this gene in all samples and subsequently dividing the resulting value by the standard deviation of the GEX of this gene in all samples.
3. The method *PCA* used the function *prcomp()* of the *stats* package of R to convert the possibly correlated raw gene expression features into a set of linearly uncorrelated principal component features. The first 10 principal components were chosen as a dimensionality-reduced feature set for the model.
4. The option *none* included the raw gene expression features without any preprocessing for the purpose of baseline comparison.

#### Feature combination

All model pipelines in this study were based on gene expression data only, since the patient data sets that were chosen for this study did not contain other molecular features.

#### BlackBox filtering

For the final step in the model development, seven different algorithms were chosen to train a model that predicts the drug response of cell lines based on baseline gene expression profiles:

1. The method *linear* used the function *lm()* of the *stats* package of R to fit a linear model to the training data.
2. The method *lasso* [42] fitted a generalized linear model with lasso penalty (*α* = 1) and a regularization parameter λ determined by a 10-fold cross-validation.
3. The method *elasticnet* [42, 43] fitted a generalized linear model with an elastic net mixing parameter of *α* = 0.5 and a regularization parameter λ determined by a 10-fold cross-validation
4. The method *rf* [44] used a random forest algorithm by [45] to fit the training data with 500 trees.
5. The method *rf_ranger* [46] established a fast implementation of a random forest model on the training data by training 10,000 trees of unlimited depth.
6. The method *ridge* [47] fitted a linear ridge regression model to the data, where the ridge parameter was chosen automatically based on a method by Cule et al. [48].
7. The method *svm* [49] trained a support vector machine with a radial kernel on the training data.

#### Validation

For the investigation of the performance of the different modeling pipelines, the area under the receiver operating curve between the true and the predicted patient classes was calculated using the *pROC* package [50].

### Statistics

#### Significance testing

In order to investigate whether two distributions were significantly different from each other the function *t.test()* of the *stats* package of R applied a two-sided student’s t-test to the two distributions.

#### Enrichment analysis

In order to investigate the enrichment of certain model settings in the overall distribution of model performances with a hypergeometric function, the method *phyper()* of the *stats* package of R was applied to the data.

#### Receiver operating curves

Receiver operating curves were calculated with the *roc()* function of the *pROC* package [50] and plotted with the *ggroc()* function of the *ggplot2* package [51].

#### Correlation

In order to determine the linear correlation of the performance distributions, the method *pearson* measured the Pearson correlation with the function *cor()* of the *stats* package of R.

#### Gene labeling

Within the context of collecting the different data sets and enforcing them into the same ForeseeObject formats for the *FORESEE* R package [19], the *biomaRt* package [22, 23] was applied to convert all gene labels into Entrez IDs.

### Plotting

#### Heatmaps

Heatmaps were plotted with the *heatmap.2()* function of the R package *gplots* [52].

#### Histograms

All histograms were plotted with the *hist()* function of the *graphics* package of R.

#### Violin plots

Violin plots were created using the *ggplot() + geom_violin()* function of the *ggplot2* package [51].

#### Correlation

The correlation plot was created using the *corrplot.mixed()* function of the *corrplot* package [53].

## Supporting information

S1 Fig FORESEE model performance for other cancer patient data sets. Portrayal of translational models that used the FORESEE package to train models on GDSC cell line data and subsequently predicted the drug response of patients from GSE18864, GSE51373, GSE33072 Erlotinib cohort, GSE33072 Sorafenib cohort, GSE9782 GLP96 cohort and GSE9782 GLP97 cohort. The settings for the respective best modeling pipelines can be found in Table 1. The patient responses were binarized as described in the paragraph “Patient response preprocessing”. (A) Receiver operating curves of the best models. (B) Distinction of true responders and non-responders obtained from the best FORESEE models, including p-values from t-tests. (C) Performance distributions of all 3,920 model pipelines.
(TIF)

S2 Fig Characterization of the underlying data structure of the training and test data sets. (A) Distributions of the gene expression values of the cell line training data sets. (B) Distributions of the gene expression values of the patient data sets implemented as ForeseeTest objects. (C) The cell line training data sets’ composition of tissues of origin. Distributions are shown for seven different patient data sets: GSE6434, GSE18864, GSE51373, GSE33072 Erlotinib cohort, GSE33072 Sorafenib cohort, GSE9782 GLP96 cohort and GSE9782 GLP97 cohort.
(TIF)

S3 Fig Violin plots of the FORESEE performances for cell2cell modeling. Distributions of the performances of 3,920 cell2cell models trained on GDSC cell line data for six different drugs: Docetaxel, Cisplatin, Erlotinib, Paclitaxel, Sorafenib and Bortezomib. Each violin plot shows the performance distribution of 3,920 modeling pipelines trained with cell response information of the respective drug. The data represents the mean performance resulting from a 5-fold cross-validation.
(TIF)

S4 Fig Boxplots of FORESEE performances for cell2cell modeling and cell2patient modeling. Boxplots showing the performance distribution of all 3,920 FORESEE pipelines on the *in vitro* validation set (red), the *in vitro* test set (yellow) and the patient data set (medium blue), versus the performance distribution of a subset of FORESEE pipelines on that data set, which were determined by choosing the 300 best pipelines from the *in vitro* validation set for six different drugs: Docetaxel, Cisplatin, Erlotinib, Paclitaxel, Sorafenib and Bortezomib.
(TIF)

S1 Table Pipeline settings for the drug specificity analysis. 100 random FORESEE pipelines were chosen and then trained on all 266 available drugs in the GDSC database to predict the patient data sets.
(XLSX)

S1 File Collection of all FORESEE model settings and results. The folder includes all relevant FORESEE model settings and modeling results presented in this manuscript.
(ZIP)
