# Supplementary figures and images for: Methodological challenges in translational drug response modeling in cancer: A systematic analysis with FORESEE
Source: PLoS Comput Biol. 2020 Apr 20;16(4):e1007803. doi: 10.1371/journal.pcbi.1007803 (PMC7192505; doi:10.1371/journal.pcbi.1007803)

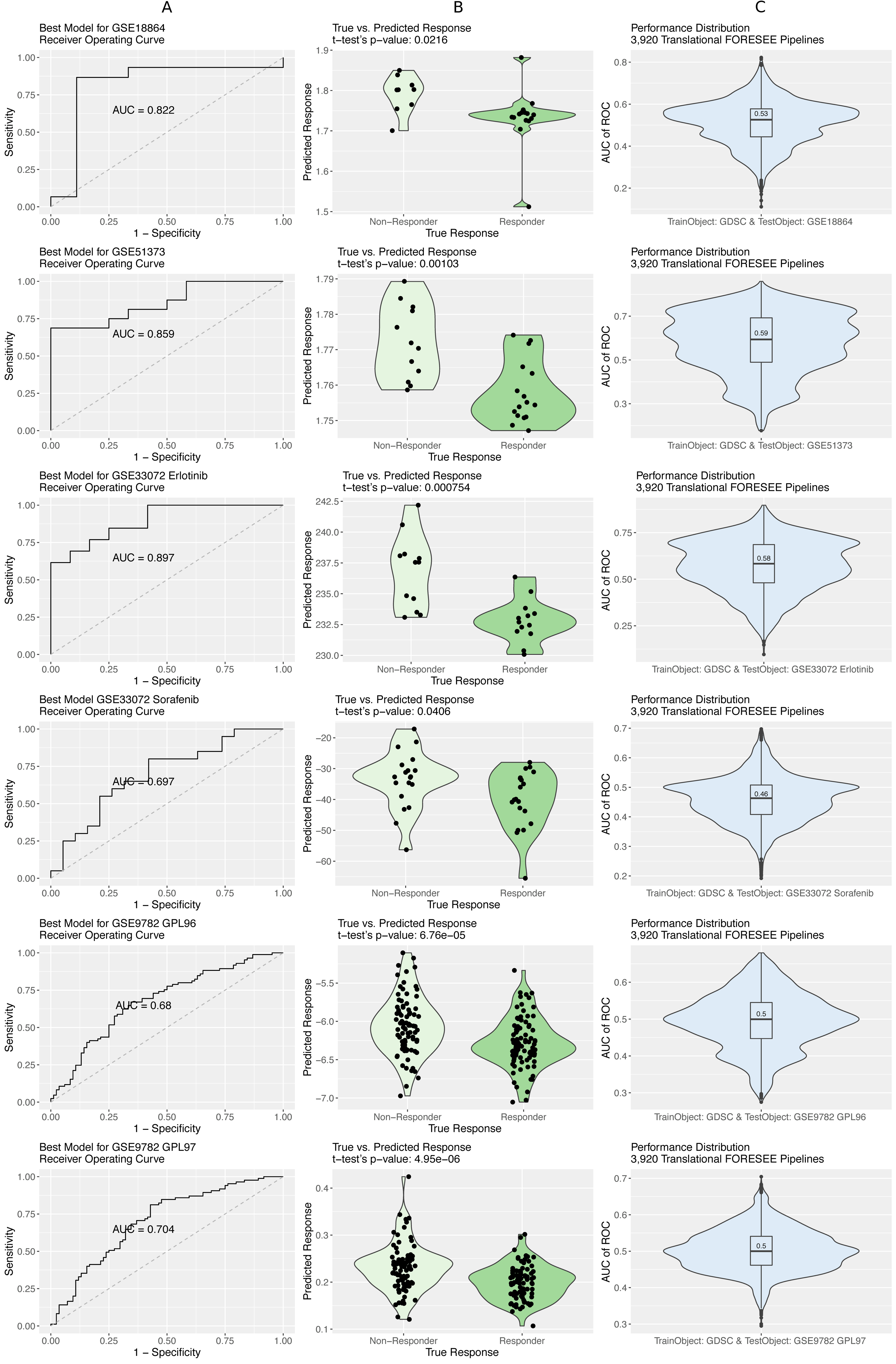

Supplement: S1 Fig — Portrayal of translational models that used the FORESEE package to train models on GDSC cell line data and subsequently predicted the drug response of patients from GSE18864, GSE51373, GSE33072 Erlotinib cohort, GSE33072 Sorafenib cohort, GSE9782 GLP96 cohort and GSE9782 GLP97 cohort. The settings for the respective best modeling pipelines can be found in Table 1. The patient responses were binarized as described in the paragraph “Patient response preprocessing”. (A) Receiver operating curves of the best models. (B) Distinction of true responders and non-responders obtained from the best FORESEE models, including p-values from t-tests. (C) Performance distributions of all 3,920 model pipelines. (TIF) [file pcbi.1007803.s001.tif]

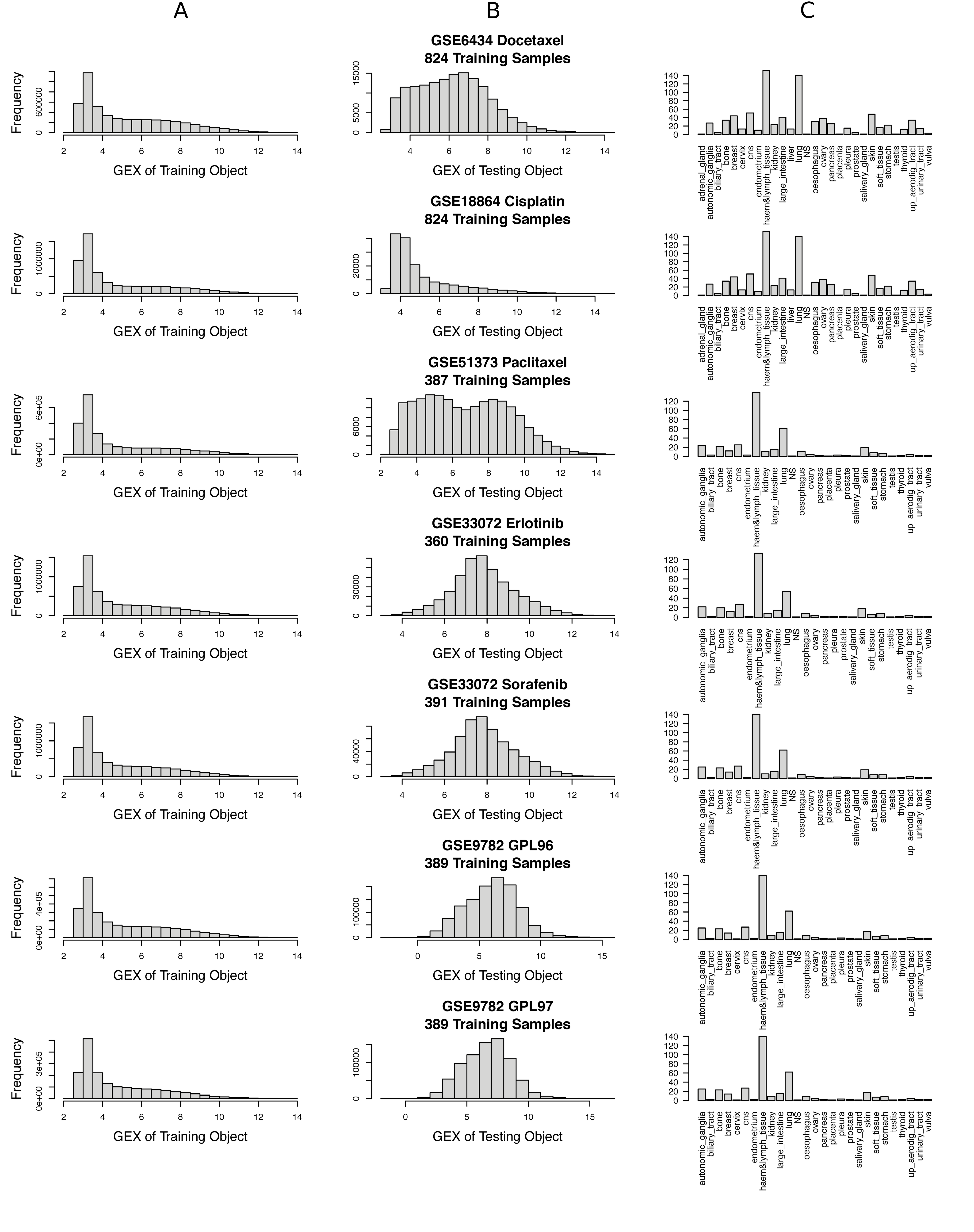

Supplement: S2 Fig — (A) Distributions of the gene expression values of the cell line training data sets. (B) Distributions of the gene expression values of the patient data sets implemented as ForeseeTest objects. (C) The cell line training data sets’ composition of tissues of origin. Distributions are shown for seven different patient data sets: GSE6434, GSE18864, GSE51373, GSE33072 Erlotinib cohort, GSE33072 Sorafenib cohort, GSE9782 GLP96 cohort and GSE9782 GLP97 cohort. (TIF) [file pcbi.1007803.s002.tif]

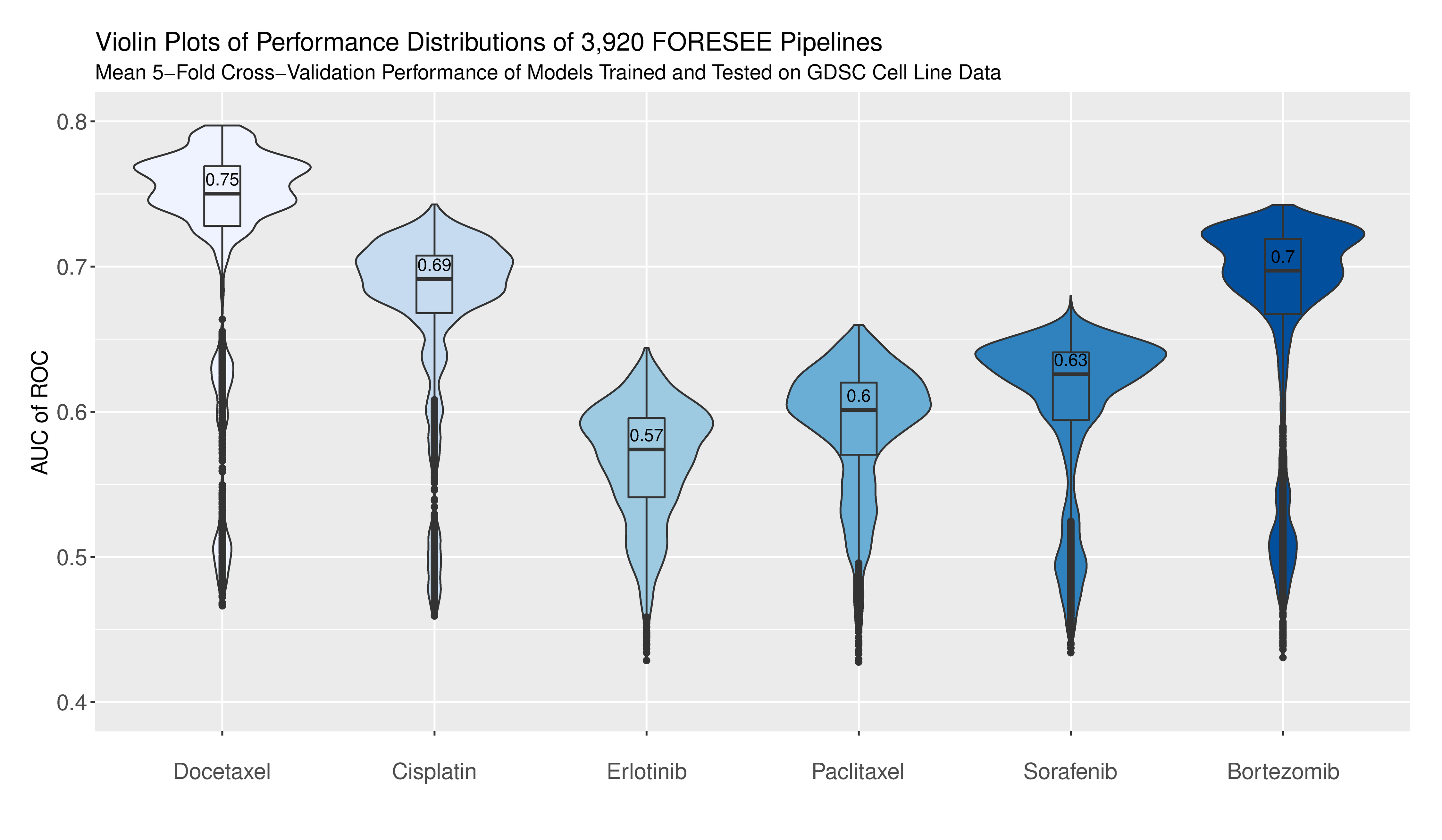

Supplement: S3 Fig — Distributions of the performances of 3,920 cell2cell models trained on GDSC cell line data for six different drugs: Docetaxel, Cisplatin, Erlotinib, Paclitaxel, Sorafenib and Bortezomib. Each violin plot shows the performance distribution of 3,920 modeling pipelines trained with cell response information of the respective drug. The data represents the mean performance resulting from a 5-fold cross-validation. (TIF) [file pcbi.1007803.s003.tif]

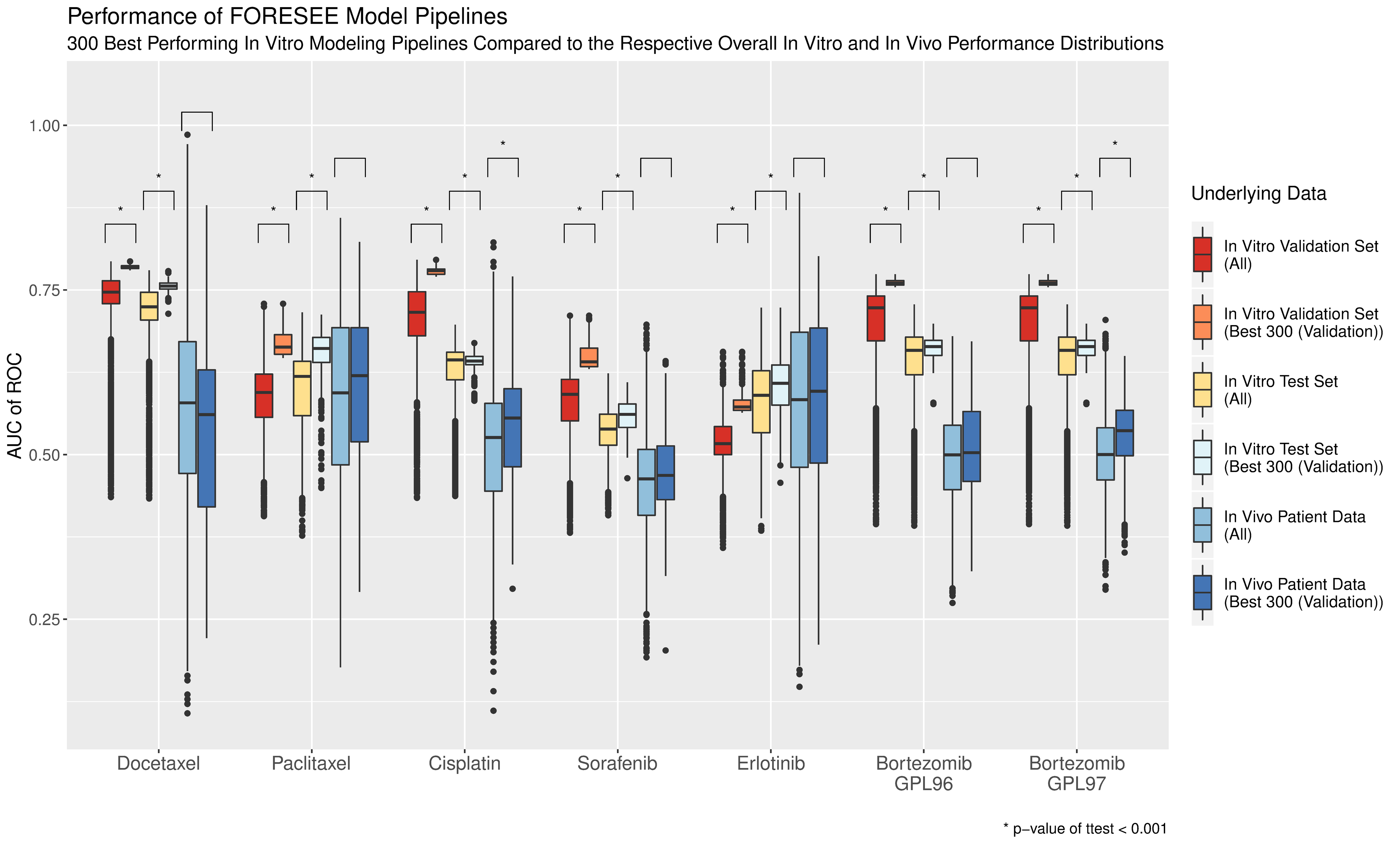

Supplement: S4 Fig — Boxplots showing the performance distribution of all 3,920 FORESEE pipelines on the in vitro validation set (red), the in vitro test set (yellow) and the patient data set (medium blue), versus the performance distribution of a subset of FORESEE pipelines on that data set, which were determined by choosing the 300 best pipelines from the in vitro validation set for six different drugs: Docetaxel, Cisplatin, Erlotinib, Paclitaxel, Sorafenib and Bortezomib. (TIF) [file pcbi.1007803.s004.tif]
